# Pulmonary [^18^F]FDG uptake demonstrates phenotypic PET behaviour in non-specific interstitial pneumonitis and refines risk stratification

**DOI:** 10.1007/s00259-026-08052-5

**Published:** 2026-07-25

**Authors:** Hannah Torlot, Balaji Ganeshan, Thida Win, Helen Garthwaite, Luke R. Hoy, Darren Walls, Raymond Endozo, Robert I. Shortman, Francesco Fraioli, Nicholas J. Screaton, Joanna C. Porter, Ashley M. Groves

**Affiliations:** 1 UCL Respiratory, University College London, London, UK; 2 Interstitial Lung Disease Service, Department of Respiratory Medicine, University College London Hospitals, London, UK; 3 NIHR UCLH Biomedical Research Centre, London, UK; 4 Institute of Nuclear Medicine, University College London /Hospital, 5th Floor, 235 Euston Road, London, NW1 2BU UK; 5 Respiratory Medicine, Lister Hospital, Stevenage, UK; 6 Department of Radiology, Papworth Hospital, Cambridge, UK

**Keywords:** Nonspecific Interstitial Pneumonitis, Interstitial Lung Disease, Positron Emission Tomography, Computed Tomography, Prognosis

## Abstract

**Rationale:**

Effective prognostic biomarkers in Non-specific Interstitial Pneumonitis (NSIP) are limited.

**Objective:**

To investigate the potential of [^18^F]FDG PET/CT to predict mortality in NSIP and to assess its relationship with physiological indices and the Interstitial Lung Disease – Gender-Age-Physiology (ILD-GAP) score.

**Methods:**

Ninety-six patients with a multidisciplinary team-confirmed diagnosis of NSIP (40 male, 56 female; mean age 59.8 ± 9.8 years) underwent baseline [^18^F]FDG PET/CT and 83 patients completed full pulmonary function testing. Pulmonary uptake of [^18^F]FDG was quantified using region-of-interest analysis as the maximum uptake in diseased lung (SUV_max_) and the background uptake in visually normal lung (SUV_min_). A target-to-background (TBR) ratio was calculated as SUV_max_ /SUV_min_. Kaplan-Meier survival analysis examined associations between the [^18^F]FDG PET/CT metrics, the pulmonary function tests and ILD-GAP scores. Stepwise forward Wald multivariable Cox regression assessed independence of the significant [^18^F]FDG PET/CT parameters from the ILD-GAP index. The modified ILD-GAP (mGAP) models were generated by incorporating the [^18^F]FDG PET/CT data into the ILD-GAP index.

**Results:**

65 patients died during a mean follow up of 83.2 ± 48.2 months. Mean ± SD SUV_max,_ SUV_min_ and TBR were 3.2 ± 1.2, 0.6 ± 0.3 and 6.0 ± 2.4, respectively. Mortality was associated with higher SUV_max_ (median cutoff ≥ 2.9, *p* = 0.038) and higher SUV_min_ (optimised cutoff ≥ 0.675, *p* < 0.001), whereas TBR was not prognostic (*p* = 0.48). Multivariable Cox regression confirmed median SUV_max_ as independent of ILD-GAP index (*p* = 0.031), although optimised SUV_min_ was not independent. Incorporating either parameter into a modified ILD-GAP model improved survival stratification compared with ILD-GAP index alone (*p* < 0.01, log-rank test).

**Conclusion:**

High pulmonary [^18^F]FDG PET/CT uptake predicts increased mortality in NSIP. Median SUV_max_ is an independent predictor of mortality and adding [^18^F]FDG PET/CT parameters to the ILD-GAP index enhances prognostic discrimination. These findings support a disease-specific interpretation of [^18^F]FDG PET/CT metrics in ILD, where SUV_max_ and SUV_min_ are dominant prognostic markers in diffuse NSIP.

## Abstract (highlights)

**Key results:**

In patients with Non-specific Interstitial Pneumonitis (N=96), mortality was associated with higher pulmonary [^18^F]FDG PET/CT uptake in both diseased lung (SUV_max_, *p*=0.038) and normal-appearing lung (SUV_min_, optimised threshold, *p*<0.001), whereas the target-to-background ratio (SUV_max_/SUV_min_) was not prognostic (*p*=0.48). Multivariable Cox analysis confirmed median SUV_max_ was independent of the ILD-GAP index (*p*=0.031). Although SUV_min_ was not independent, incorporating SUV_max_ or SUV_min_ into a modified ILD-GAP score improved survival stratification (*p*<0.01).

**Summary statement:**

Median SUV_max_ and optimised SUV_min_ cutoffs are prognostic in NSIP and this contrasts with Idiopathic Pulmonary Fibrosis where TBR dominates. Combining [^18^F]FDG PET/CT parameters with ILD-GAP modelling improves survival prediction in NSIP.

## Introduction

Interstitial lung disease (ILD) is a heterogeneous group of disorders characterised by varying degrees of inflammation and fibrosis. Among them, nonspecific interstitial pneumonia (NSIP) has emerged over the past three decades as a distinct clinicopathological entity, distinguishable by its radiological features, clinical associations, and variable clinical course [1]. On high-resolution CT (HRCT), NSIP typically shows bilateral, symmetrical ground-glass opacities with basal reticulation, traction bronchiectasis, and volume loss [1].

NSIP may occur idiopathically or in association with a range of conditions, including connective tissue disease (particularly systemic sclerosis), chronic hypersensitivity pneumonitis, drug toxicity, and slowly resolving diffuse alveolar damage [2]. Although its overall prognosis is generally more favourable than that of usual interstitial pneumonia (UIP), predicting individual outcomes remains challenging [3]. Some patients improve or remain stable with treatment, while others progress to end-stage fibrosis. Moreover, radiological NSIP can represent early or “fine” fibrosis rather than inflammation and may therefore signal the onset of progressive pulmonary fibrosis (PPF), including idiopathic pulmonary fibrosis (IPF) [4].

Currently, lung transplantation remains the only curative option for PPF [5], although antifibrotic therapies are approved for IPF and selected non-IPF progressive fibrosing ILDs [6]. In practice however, many patients with NSIP are treated empirically with immunosuppression while clinicians await evidence of progression - potentially delaying antifibrotic treatment in those who might benefit, while exposing others to unnecessary therapy. This underscores the need for non-invasive biomarkers that can identify high-risk patients early and guide treatment decisions.

^18^F-fluorodeoxyglucose positron emission tomography/computed tomography ([^18^F]FDG PET/CT) can non-invasively investigate cellular metabolism in vivo and previous work has shown increased pulmonary uptake of [^18^F]FDG in IPF and other ILDs, correlating with disease severity and mortality [7–9]. In IPF, a high target-to-background ratio (TBR) - the ratio of maximum to minimum standardised uptake value (SUV_max_/SUV_min_) - is a powerful predictor of poor outcome [10, 11]. Conversely, in systemic sclerosis-associated ILD (SSc-ILD), we recently demonstrated that high SUV_max_, high SUV_min_, and low TBR were each associated with worse survival, reflecting a more diffuse metabolic pattern [12]. Increased [^18^F]FDG uptake has also been linked to more severe disease [9, 13] and treatment response [14] in NSIP, although robust prospective studies remain limited.

We therefore undertook this study to determine whether the [^18^F]FDG PET/CT phenotype observed in SSc-ILD (high SUV_max_, high SUV_min_, low TBR) extends to other forms of NSIP and to contrast these findings with the typical high TBR pattern seen in IPF. Establishing such disease-specific imaging signatures could support the use of [^18^F]FDG PET/CT uptake as a quantitative imaging biomarker for risk stratification and treatment planning in diffuse parenchymal lung disease.

The primary objective of this study was to evaluate the prognostic value of [^18^F]FDG PET/CT metrics (SUV_max_, SUV_min_, and TBR) in patients with NSIP and to determine whether these imaging parameters provide information independent of physiological indices. A secondary aim was to explore whether combining [^18^F]FDG PET/CT parameters with the ILD Gender-Age-Physiology (GAP) model could improve risk stratification, thereby recommending a disease-specific approach to [^18^F]FDG PET/CT interpretation approach across different ILD subtypes.

## Methods

### Patients

Consecutive patients with a multidisciplinary team (MDT) diagnosis of NSIP at University College London Hospitals were prospectively recruited for baseline [^18^F]FDG PET/CT scans and lung function assessment. The study was approved by the London-Harrow Research Ethics Committee (REC reference: 06/Q0505/22) and all participants provided written informed consent. A proportion of participants were included in an earlier report focusing on SSc-ILD [12], but the current study extends the administrative follow-up period for these patients by 47 months and presents new analyses not previously published.

**Fig. 1:**
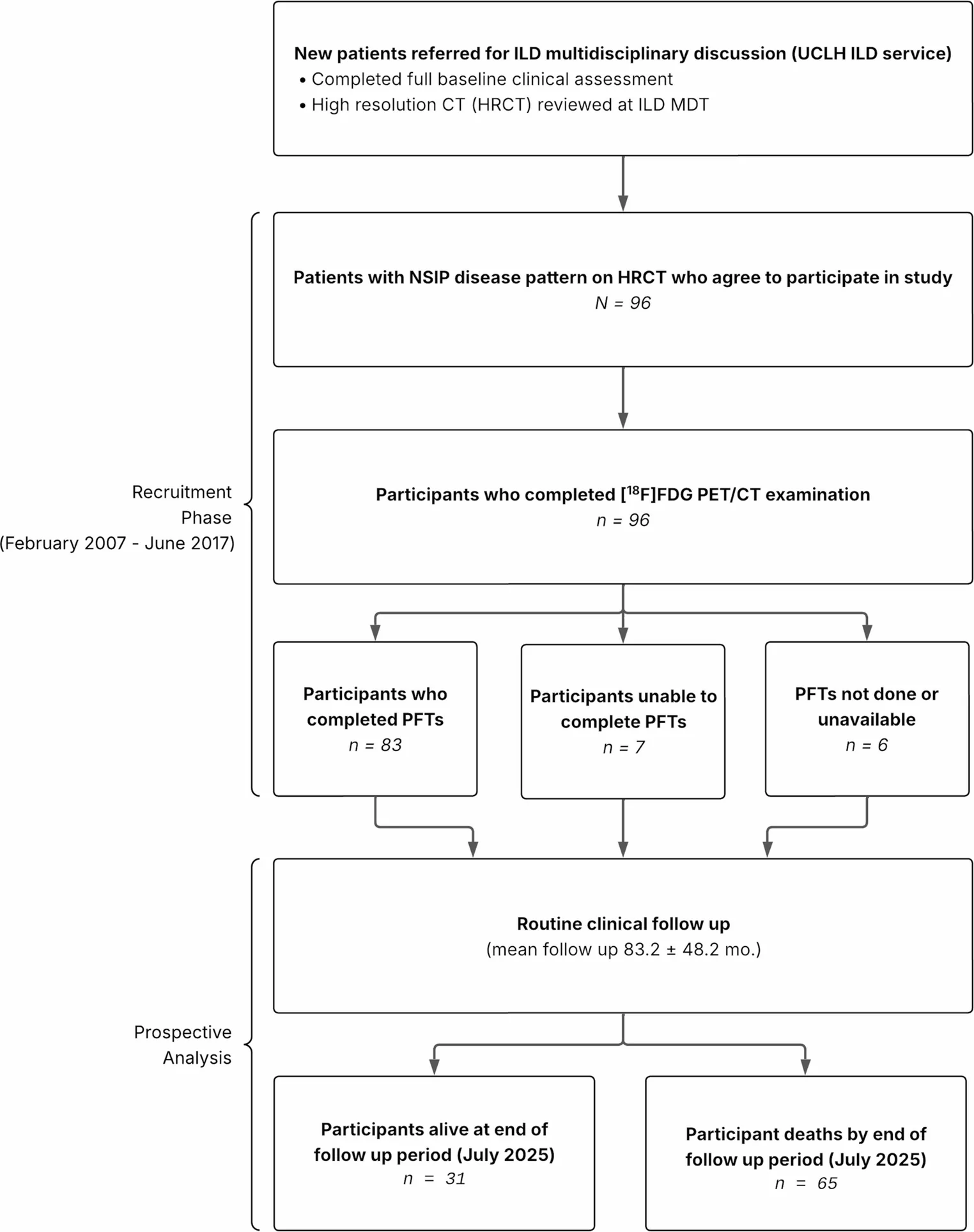
Flow diagram detailing the recruitment and analysis phases for patients included in this prospective observational study (*N* = 96). Patients followed for a mean 83 months, during which 65 deaths occurred

All patients had a clinico-radiological diagnosis of NSIP following an HRCT scan and a full clinical assessment that included baseline pulmonary function tests (forced vital capacity (FVC), forced expiratory volume in 1 s (FEV₁), and transfer factor and coefficient for carbon monoxide (TL_CO_ and K_CO_), successfully performed by 83 patients). Patients with infection or neoplasia were excluded on clinical and radiological grounds. The recruitment process and subsequent observational phases are shown in Fig. 1.

### Patient follow-up

Follow-up duration was defined as the time from the date of the baseline [^18^F]FDG PET/CT scan to all-cause death or lung transplant, with patients censored at the date of their last known contact with medical services or at the end of data collection (July 2025). Survival status was confirmed using hospital records, electronic databases, primary care records, and telephone contact where required.

### [^18^F]FDG PET/CT acquisition and analysis

#### Image acquisition

All imaging was performed on the same PET/CT scanner (VCT PET/64-detector CT system; GE HealthCare). Patients fasted for at least six hours before receiving an intravenous injection of approximately 200 MBq of [^18^F]FDG. After a 60-minute uptake period, a low dose CT scan was performed of the thorax for attenuation correction and anatomical localisation, followed immediately by a PET emission scan of the same region (8 min per bed position).

#### Image analysis

[^18^F]FDG PET/CT datasets were reviewed on a dedicated workstation by a PET Radiologist and a senior PET Technologist, each with > 10 years’ experience in quantifying pulmonary [^18^F]FDG uptake on PET/CT. Analysis was performed under the supervision of a dual-trained Radiologist/Nuclear Medicine Physician (> 10 years’ experience). CT and HRCT images were reviewed by a Thoracic Radiologist with expertise in ILD, blinded to the PET emission data.

**Fig. 2:**
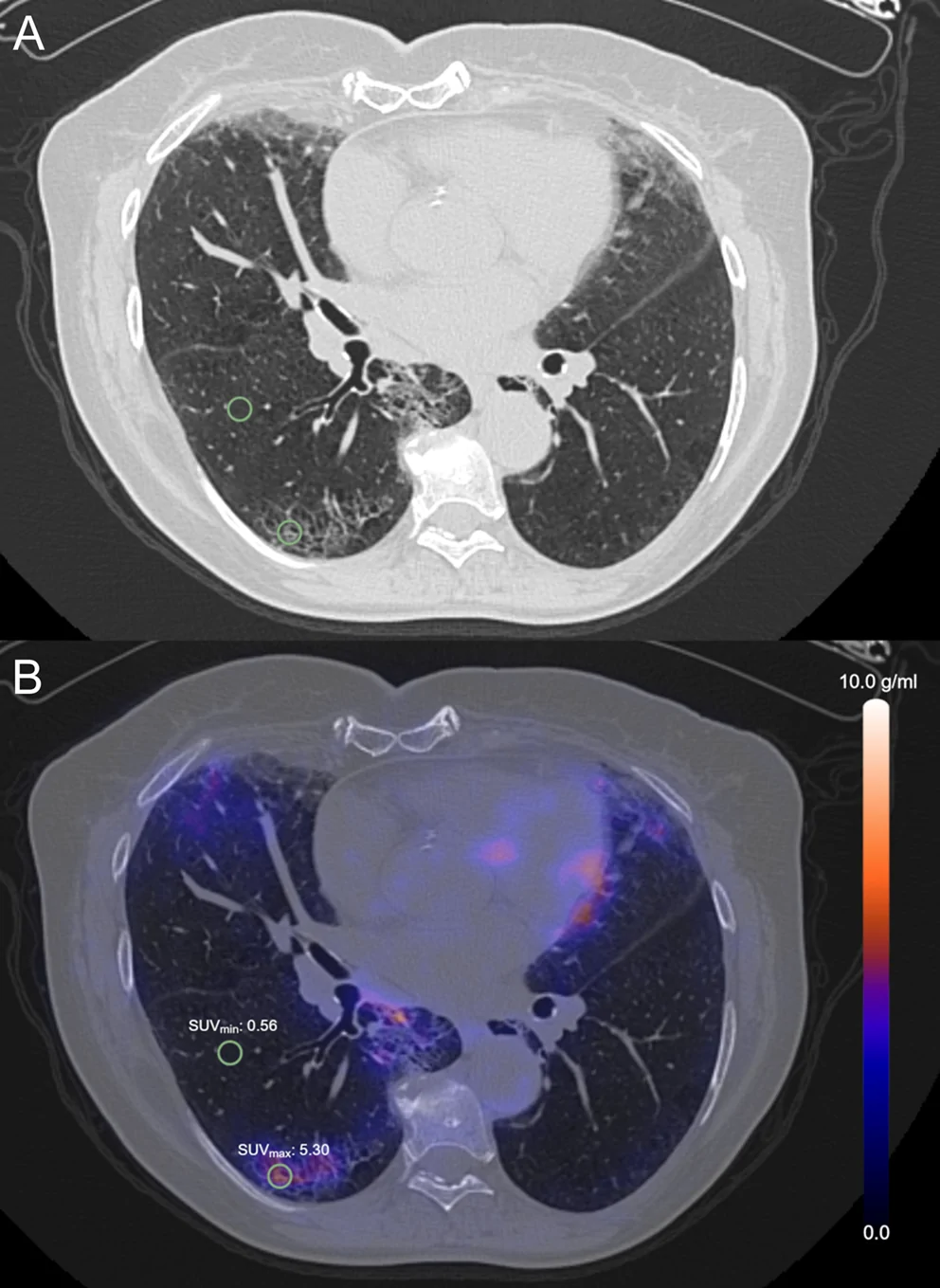
CT scan (**A**) and co-registered [^18^F]FDG PET/CT (**B**) of a patient with non-specific interstitial pneumonia, showing region of interest placement measuring maximal pulmonary [^18^F]FDG uptake (SUV_max_) and background uptake in unaffected lung (SUV_min_)

Quantitative analysis was performed using a region-of-interest (ROI) method on co-registered axial images (Fig. 2). SUV_max_ was defined as the highest pixel value within a 2-dimensional ROI placed over the area of greatest pulmonary [^18^F]FDG PET/CT uptake. SUV_min_ was defined as the lowest pixel value within an ROI drawn over morphologically normal-appearing lung parenchyma as confirmed by the Thoracic Radiologist with reference to the prior HRCT. TBR was calculated as SUV_max_ /SUV_min_ and therefore reflected the contrast between metabolically active and unaffected lung.

The HRCT datasets were reviewed independently by the Thoracic Radiologist who classified the ILD pattern of disease as NSIP according to established radiological criteria.

### ILD-GAP calculation

The ILD-GAP model [15] is based on four variables: gender, age and two lung physiology measures (FVC and TLCO). The ILD-GAP score was calculated according to established methodology and ranged from 0 to 8 with higher scores indicating increasing mortality risk. The ILD-GAP score was converted to ordinal ILD-GAP indices I–IV (I = 0–1, II = 2–3, III = 4–5, IV = 6–8) where increasing rank corresponds to reduced survival probability.

### Statistical analysis

All statistical analyses were performed using SPSS (version 31.0; IBM, Armonk, NY, USA). Continuous variables are presented as mean ± standard deviation, and categorical variables as counts or percentages. A two-tailed *p* < 0.05 was considered statistically significant.

#### Univariate survival analysis

Associations between [^18^F]FDG PET/CT parameters (SUV_max_, SUV_min_, TBR), pulmonary function tests (FEV₁, FVC, TLCO, KCO) and ILD-GAP (score and index) were explored using Kaplan-Meier survival analysis with log-rank testing. Both median and optimised cut off methodologies (the latter defined by the threshold yielding the lowest log-rank p-value) were applied to SUV_max_, SUV_min_, and TBR to define “good” and “poor” prognostic groups. Kaplan-Meier curves were constructed to display survival probabilities for each group.

#### Multivariable Cox regression

Stepwise forward Wald Cox proportional-hazards models were used to identify independent predictors of mortality among the variables that were significant in univariate analysis and to assess interactions between [^18^F]FDG PET/CT metrics and physiological measures.

#### Modelling [^18^F]FDG PET/CT data combined with ILD-GAP in a modified ILD-GAP score

To evaluate whether [^18^F]FDG PET/CT data improved physiological risk stratification, a modified ILD-GAP score (mGAP) was generated by using the [^18^F]FDG PET/CT data and prognostic cutoffs to create an additional binary variable to the original ILD-GAP score. This variable was coded as “1” or “0” for adverse (SUV_max_ ≥ median; SUV_min_ ≥ optimised) or favourable prognosis, respectively. The resulting mGAP scores ranged from 0 to 9 and were incorporated into the ILD-GAP index as follows: I = 0–2, II = 3–4, III = 5–6, IV = 7–9.

#### Correlation analyses

Correlations between [^18^F]FDG PET/CT parameters SUV_max_, SUV_min_ and TBR were evaluated using Pearson or Spearman coefficients, as appropriate.

## Results

### Cohort characteristics

Between February 2007 and June 2017, 96 patients with NSIP were recruited (Table 1). All patients underwent [^18^F]FDG PET/CT and 83 patients had recorded pulmonary function testing (7 patients were unable to perform the TLco measurement due to reduced lung capacity and data was unavailable for 6 patients). During a mean follow up of 83 months, 65 patients died.

**Table 1:** Patient characteristics (*N* = 96) at baseline with pulmonary function test results and high-resolution Computed Tomography findings. Values expressed as mean ± standard deviation for continuous variables and frequencies with percentages (%) for qualitative variables. Pulmonary function tests expressed as percentage predicted

Characteristic	Patients (*N* = 96)
Sex (M: F)	40:56
Mean age, years (± SD)	60.6 ± 9.9 years
Lung Function Tests	
FVC (% predicted)	73.52 ± 20.39
FEV_1_ (% predicted)	70.20 ± 18.78
K_CO_ (_min_^−1^)	65.41 ± 17.71^‡^
TL_CO_ (mmol _min_^−1^ kPa^− 1^)	41.94 ± 15.83^‡^
Underlying diagnosis, n (%)	
Systemic sclerosis	40 (41.7%)
Rheumatoid arthritis	11 (11.5%)
Undifferentiated connective tissue disease	8 (8.3%)
Antisynthetase syndrome	6 (6.3%)
Sjӧgren’s	2 (2.0%)
Drug reaction	2 (2.1%)
Langerhans cell histiocytosis	1 (1.0%)
Vasculitis	1 (1.0%)
Other	7 (7.3%)
Idiopathic NSIP	18 (18.8%)

*FVC *Forced vital capacity, *FEV*_*1*_ Forced expiratory volume in 1 s, *K*_*co*_ Carbon monoxide transfer coefficient, *TL*_*co*_ Lung transfer factor carbon monoxide

‡ *n* = 83 as 13 patients were either unable to complete gas transfer measurements or data was unavailable

### [^18^F]FDG PET/CT and physiological predictors of outcome

[^18^F]FDG PET/CT metrics and physiological measurements together with their univariate associations with survival are summarised in Table 2.

**Table 2:** Associations between [^18^F]FDG PET/CT parameters, lung function indices, and ILD-GAP variables with overall survival in NSIP (N = 96), using log-rank testing at both median and optimised cutoffs (the latter determined by minimising the log-rank *p*-value). Values show the number of patients in poor- and good-prognosis groups for each parameter and corresponding *p*-values. GAP score and stage are expressed as categorical thresholds. Significant results (*p* < 0.05) are highlighted in bold

Parameter	Median	Poor	Good	*p*-value	Optimised	Poor	Good	*p*-value
SUV_max_	≥ 2.9	51	45	**0.027**	≥ 2.975	45	51	**0.007**
SUV_min_	≥ 0.51	48	48	0.123	≥ 0.675	26	70	**< 0.001**
TBR	< 5.44286	48	48	0.476	< 2.86607	9	87	**0.022**
FVC	< 73.2	48	48	**0.018**	< 58.35	26	70	**0.008**
FEV1	< 68	46	49	0.581	< 66.5	40	55	**0.044**
TLC	< 67.5	17	17	**0.021**	< 67.5	17	17	**0.021**
KCO^‡^	< 78	38	42	0.063	< 64.85	22	58	**0.002**
TLCO^‡^	< 47.7	41	42	**0.003**	< 48.8	46	37	**0.002**
Gender					Male	40	56	0.557
Age	≥ 62.0	49	47	0.140	≥ 70.5	15	81	**< 0.001**
GAP score	≥ 1	57	33	**0.006**	≥ 2	28	62	**0.001**
GAP stage					≥II	28	62	**0.001**

*FVC* Forced vital capacity, *FEV*_*1*_ Forced expiratory volume in 1 s, *TLC* Total lung capacity, *K*_*co*_ Carbon monoxide transfer coefficient, *TL*_*co*_ Lung transfer factor carbon monoxide

‡ *n* = 83 as 13 patients were either unable to complete gas transfer measurements or data was unavailable

**Fig. 3:**
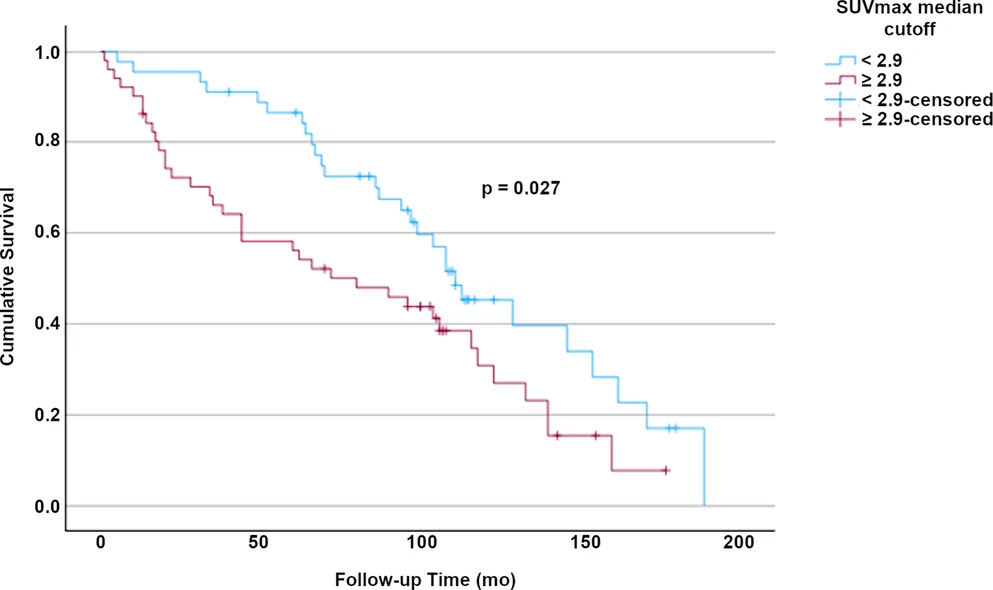
Kaplan-Meier survival curve over the 200-month follow-up period for patients with NSIP (*N* = 96) showing poorer survival among those with disease-affected lung SUV_max_ ≥2.9 (median cutoff; *p* = 0.027, log-rank test), demonstrating that elevated pulmonary metabolic activity predicts adverse outcome

**Fig. 4:**
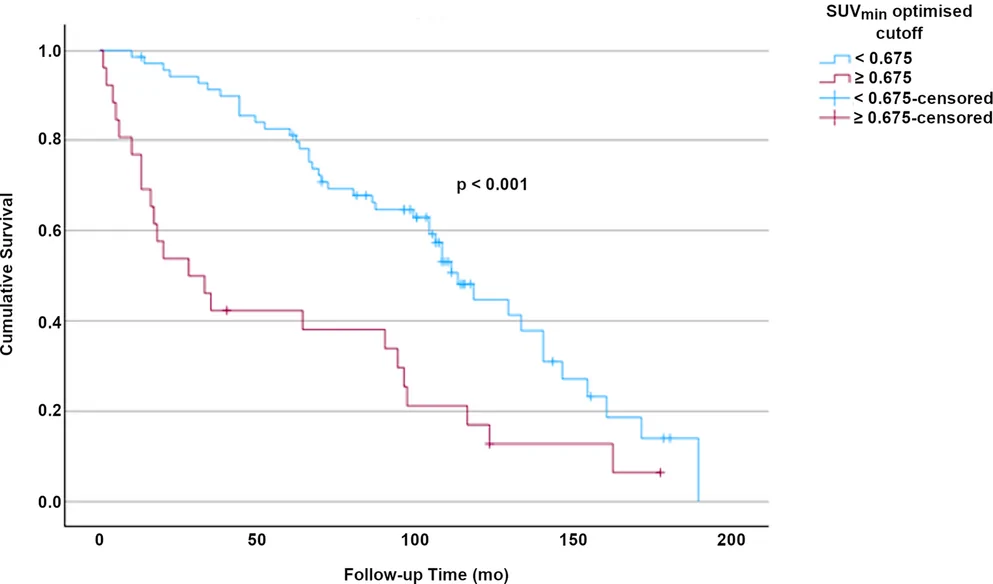
Kaplan-Meier survival curve over the 200-month follow-up period for patients with NSIP (*N* = 96) showing poorer survival among those with background parenchymal [^18^F]FDG PT/CT uptake SUV_min_ ≥0.675 (optimised cutoff; *p* < 0.001, log-rank test), demonstrating that patients with higher background parenchymal uptake had significantly poorer survival

SUV_max_ was associated with poorer outcome at the median (≥ 2.9; *p* = 0.027) and the optimised cutoffs (≥ 2.975; *p* = 0.007), confirming it as a robust survival marker across the cohort (Fig. 3). SUV_min_ was not significantly associated with poorer outcome at the median cutoff (≥ 0.51; *p* = 0.123) but was strongly prognostic at the optimised cutoff (≥ 0.675; *p* < 0.001) (Fig. 4).

TBR was not significantly associated with poorer outcome at the median cutoff (< 5.442; *p* = 0.476) but became significant at a low optimised cutoff (< 2.866; *p* = 0.022). The TBR median cutoff value showed no prognostic separation within the cohort and the trend of lower TBR and reduced survival was opposite to that previously seen in IPF and could be explained by the typically higher background [^18^F]FDG uptake in NSIP.

Significant associations with shorter survival were observed for FVC (< 73.2, median, *p* = 0.018; < 58.35, optimised, *p* = 0.008), FEV1 (< 66.5, optimised, *p* = 0.044), KCO (< 64.85, optimised, *p* = 0.002), TLCO (< 47.7, median, *p* = 0.003; < 48.8, optimised, *p* = 0.002), Age (≥ 70.5, optimised, *p* < 0.001), GAP score (> 1, median, *p* = 0.006; > 2, optimised, *p* = 0.001) and GAP index (> 2, optimised, *p* = 0.001).

These associations are summarised in Table 3 with the estimated 2-year and 5-year survival percentages for each optimised cutoff value and the median survival of each prognostic group.

**Table 3:** Estimated 2-year and 5-year survival percentages with 50% survival in months for each of the significant variables based on the optimised cutoff values for [^18^F]FDG PET/CT (SUV_max_, SUV_min_, TBR), and pulmonary function test parameters (FVC, K_CO_, TL_CO_) and ILD-GAP score and index. Estimates of median survival where applicable in months

Variable (Optimised cutoff)	Prognostic Group	2-yr Survival (%)	5-yr Survival (%)	Median Survival (mo)
SUV_max_ ≥ 2.975	Poor	69%	54%	66
SUV_max_ < 2.975	Good	96%	86%	111
SUV_min_ ≥ 0.675	Poor	54%	43%	28
SUV_min_ < 0.675	Good	94%	81%	113
TBR < 2.86607	Poor	56%	45%	28
TBR ≥ 2.86607	Good	86%	73%	108
FVC < 58.35	Poor	73%	46%	44
FVC ≥ 58.35	Good	87%	80%	111
KCO < 64.85^‡^	Poor^‡^	73%^‡^	54%^‡^	62^‡^
KCO ≥ 64.85^‡^	Good^‡^	95%^‡^	83%^‡^	118^‡^
TLCO < 48.8^‡^	Poor^‡^	78%^‡^	56%^‡^	69^‡^
TLCO ≥ 48.8^‡^	Good	98%	95%	129
GAP score ≥ 2	Poor	61%	50%	44
GAP score < 2	Good	91%	79%	111
GAP stage ≥ II	Poor	61%	50%	44
GAP stage = I	Good	92%	79%	111

*FVC* Forced vital capacity, *FEV*_*1*_ Forced expiratory volume in 1 s, *K*_*co*_ Carbon monoxide transfer coefficient, *TL*_*co*_ Lung transfer factor carbon monoxide

‡ *n* = 83 as 13 patients were either unable to complete gas transfer measurements or data was unavailable

### Multivariate Cox regression analyses assessing independence of [^18^F]FDG PET/CT markers and ILD-GAP index

In multivariable Cox models, SUV_max_ (median cutoff ≥ 2.9) remained an independent predictor of survival after adjustment for ILD-GAP index (*HR* 1.80, 95% CI 1.06–3.10, *p* = 0.031). SUV_min_ (optimised cutoff ≥ 0.675) was not independently significant when adjusted for ILD-GAP index but demonstrated synergistic behaviour when modelled as an interaction term, suggesting complementary prognostic information between SUV_min_ and physiology.

### Modelling of [^18^F]FDG PET/CT derived SUV_min_ in combined ILD-GAP analysis

**Fig. 5:**
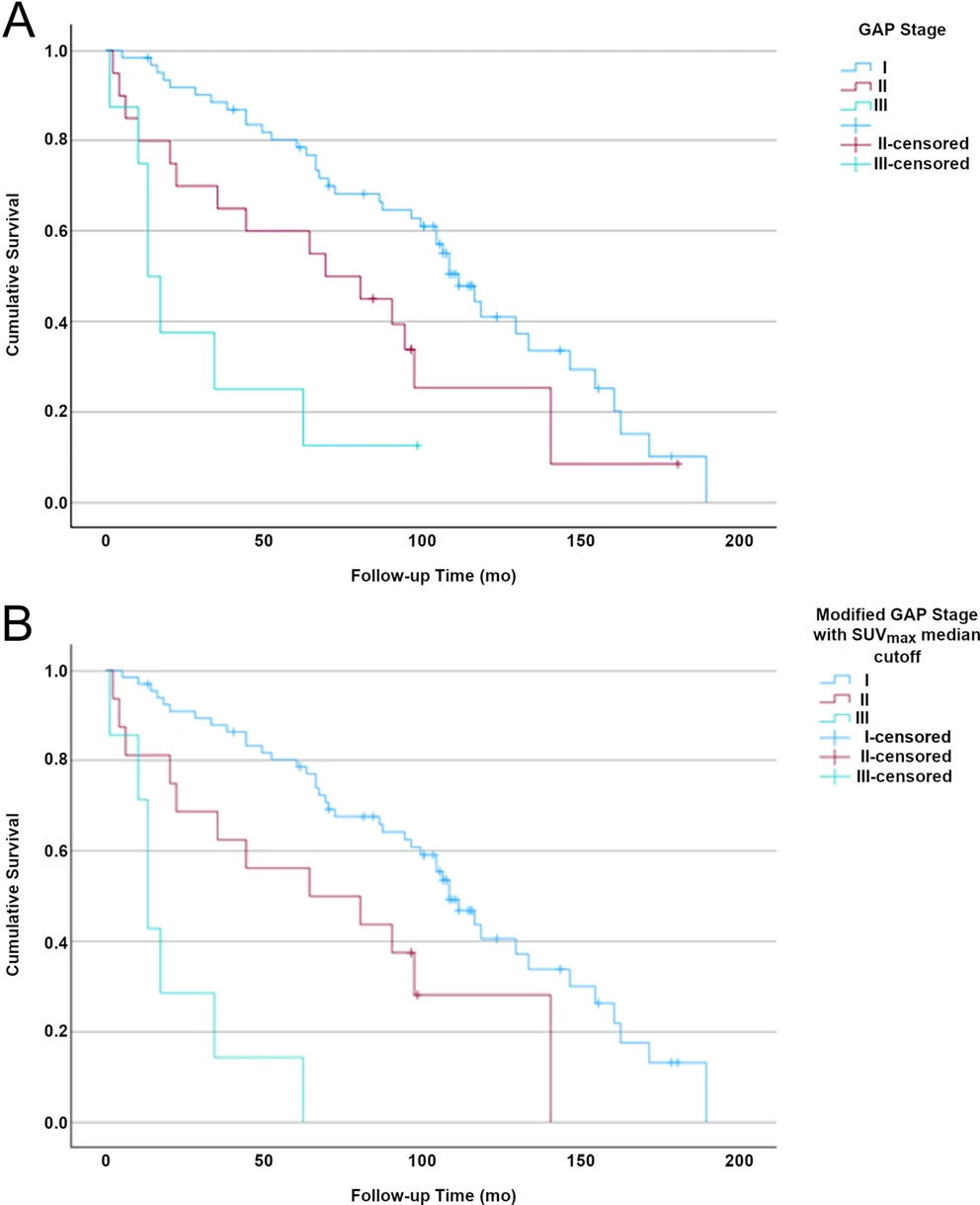
Kaplan-Meier survival curves over the 200-month follow-up for patients with NSIP (*N* = 96) stratified by ILD-GAP index (stages I-III) (**A**) and stratified by the modified ILD-GAP index (**B**) which incorporates the median SUV_max_ ≥2.9 as an adverse prognostic marker. The addition of SUV_max_ improved survival discrimination compared with the unmodified GAP (*p* < 0.01, log-rank test), demonstrating that [^18^F]FDG PET/CT derived metabolic activity provides additive prognostic value in NSIP

**Fig. 6:**
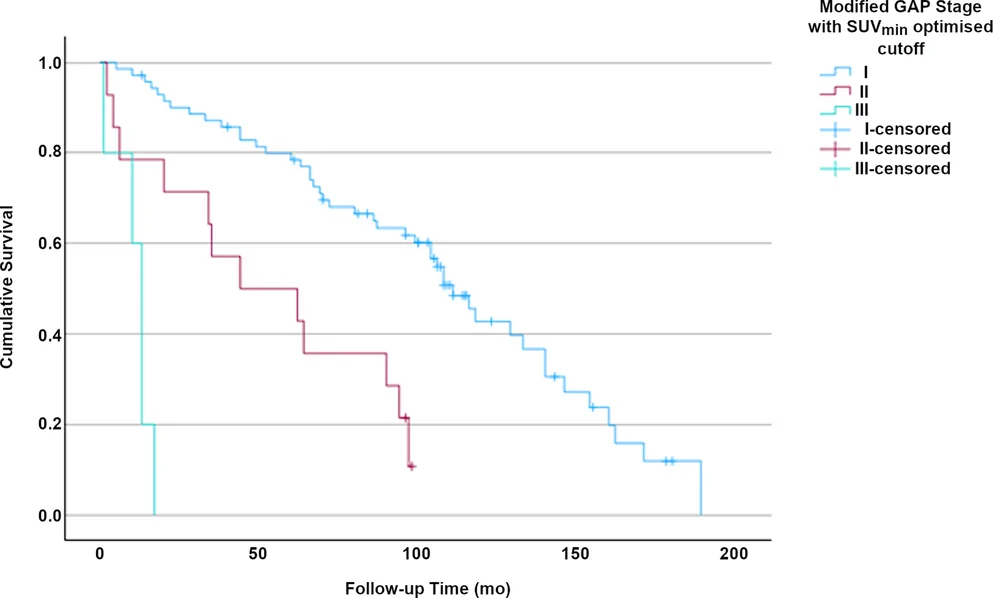
Kaplan-Meier survival curves over the 200-month follow-up for patients with NSIP (*N* = 96) stratified by the modified ILD-GAP index (stages I-III) incorporating SUV_min_ ≥0.675 as an adverse prognostic marker demonstrates a significant difference (*p* < 0.001, log-rank test)

Combining [^18^F]FDG PET/CT data with physiological indices enhanced prognostic stratification. The mGAP model incorporating the median SUV_max_ cutoff improved survival discrimination compared with the original ILD-GAP index (log-rank *p* < 0.01; Fig. 5). Although SUV_min_ was not independent of ILD-GAP index in multivariable analysis, a separate exploratory modelling using the optimised SUV_min_ cutoff instead of the median SUV_max_ cutoff also improved survival discrimination compared with the unmodified GAP model (*p* < 0.001, log-rank test; Fig. 6) supporting complementary roles for metabolic and physiological indices in NSIP.

### Correlative analysis

SUV_min_ and TBR were strongly negatively correlated (ρ = − 0.65, *p* < 0.001), confirming that high background uptake compresses TBR values which may underlie the apparent reversed prognostic behaviour in NSIP. SUV_max_ and SUV_min_ showed only a weak-to-moderate positive correlation (ρ = 0.29, *p* = 0.004), indicating that they capture distinct aspects of pulmonary metabolic activity.

In summary, this study identifies SUV_max_ as an independent [^18^F]FDG PET/CT marker of outcome in NSIP and reveals SUV_min_ may have a complementary role in establishing a foundation for a stratified, phenotype-driven approach to molecular imaging in ILD.

## Discussion

We present the largest prospective cohort to date evaluating [^18^F]FDG PET/CT in patients with NSIP. In this population, baseline pulmonary [^18^F]FDG uptake was strongly associated with survival, with high pulmonary SUV_max_, high SUV_min_ and low TBR each associated with poorer outcomes. Among these, [^18^F]FDG uptake in affected lung (SUV_max_) provided independent prognostic information beyond the ILD-GAP index, while background uptake in morphologically normal lung (SUV_min_) and the derived SUV_max_/SUV_min_ ratio (TBR) were not independently predictive. Although our optimised thresholds derived through minimum log-rank p-values were not externally validated, we have included median cut-off values as a more neutral comparator. The median SUV_max_ cut-off value remained statistically significant, reinforcing it as a valid prognostic metric, while the median SUV_min_ – although significant at the optimised threshold – was non-significant at the median cut-off.

While physiological indices such as FVC and the ILD-GAP score reflect the functional impact of disease, they do not fully capture the spatial distribution of parenchymal involvement. In this study, PET-derived metabolic activity (particularly SUV_max_) provided prognostic information independent of physiology, suggesting that metabolic imaging captures complementary aspects of disease biology. Conceptually, disease burden may reflect an interaction between anatomical extent and metabolic intensity, although CT extent was not formally quantified in this cohort and this hypothesis requires prospective validation.

The ability of treatment to modify the [^18^F]FDG PET/CT signal in NSIP remains uncertain and warrants further study, particularly given that antifibrotic therapies such as pirfenidone and nintedanib have not been shown to consistently alter FDG uptake in IPF [16].

A key observation was that TBR was not a reliable prognostic marker in NSIP unlike in IPF. This likely reflects the diffuse, relatively homogeneous background uptake characteristic of NSIP, where SUV_min_ values are higher and compress the SUV_max_/SUV_min_ ratio. IPF typically shows low background uptake with patchy areas of intense metabolic activity making TBR a more effective marker of disease as shown in previous studies.

Taken together, these findings support a disease-calibrated approach to [^18^F]FDG PET/CT interpretation in ILD. In patchy fibrotic diseases such as IPF, TBR remains informative as it accentuates regional metabolic contrasts while in more diffuse inflammatory–fibrotic processes such as NSIP or SSc-ILD, absolute [^18^F]FDG uptake measures - particularly SUV_max_, but in some cases SUV_min_ - better reflect overall disease burden and prognosis. In SSc-ILD, vascular inflammation and endothelial activation may contribute to high background uptake giving SUV_min_ additional prognostic significance. Thus, the dominant [^18^F]FDG PET/CT marker may vary by disease phenotype, reinforcing the need for a calibrated, mechanism-informed framework for molecular imaging in ILD.

These findings support the concept that the prognostic utility of [^18^F]FDG PET/CT metrics varies according to ILD phenotype, reflecting underlying biological heterogeneity that is not fully captured by physiological indices alone and may therefore provide complementary information to established clinical risk scores such as ILD-GAP. Incorporation of [^18^F]FDG PET/CT parameters into an ILD-specific prognostic framework (as illustrated by the exploratory modified GAP model) improved survival stratification in this cohort. However, this approach should be regarded as hypothesis-generating, given its derivation from a single dataset and lack of external validation. Rather than immediate clinical application, these findings may be most relevant in early-phase stratification and trial design, where [^18^F]FDG PET/CT imaging could provide a more sensitive readout of biological activity than serial physiological decline alone, potentially enabling more efficient assessment of target-engagement and therapeutic response in early phase clinical trials in ILD.

### Study limitations and future directions

This was a single-centre, modestly sized cohort of patients with NSIP with heterogeneous aetiologies, reflecting real-world complexity. Although the prognostic value of SUV_max_ and SUV_min_ was robust and independent of physiology, validation in external cohorts and standardisation of acquisition protocols will be essential. Longitudinal studies are warranted to explore how [^18^F]FDG PET/CT activity evolves with treatment and whether early metabolic changes predict subsequent physiological or radiological progression. Integrating [^18^F]FDG PET/CT metrics with molecular and quantitative CT data may also enhance phenotypic characterisation and refine outcome prediction in diffuse parenchymal lung disease.

There are several recognised technical challenges relating to PET quantification in diffuse lung disease and the nuances have been previously discussed [17, 18]. ILD causes changes in the amount of air, blood and water within the lungs depending on disease burden and activity which affects the apparent FDG uptake within the lung parenchyma. Air-fraction correction and blood-volume correction may be helpful in addressing this variability but represents additional procedural complexity that may not translate into clinical practice [19]. Likewise, methods for controlling respiratory motion through respiratory gating or shallow breathing face practical limitations around time, technology and breath-holding ability in patients with impaired lung function. The use of SUV_min_ as opposed to more conventional SUV_mean_ to quantify background lung uptake reflects the inherent heterogeneity of ILD and allowed the avoidance of metabolically active (but normal appearing lung parenchyma on high resolution CT), and increased the dynamic range from which quantitative thresholds could be derived. This methodology has shown potential to differentiate metabolism in normal appearing parenchyma in IPF patients from that in a control group [20]. Background uptake quantification with SUV_min_ is susceptible to image noise, partial volume effects and reconstruction artefacts, and further investigation is needed.

## Conclusion

In this study, SUV_max_ and SUV_min_ emerge as complementary, disease-specific [^18^F]FDG PET/CT signatures in NSIP, reflecting both focal and diffuse metabolic activity within the lung. While SUV_max_ was the dominant independent predictor in this cohort, the prognostic value of SUV_min_ in SSc-ILD has been previously established and likely reflects microvascular inflammation and endothelial activation [21]. These features may also contribute to background uptake in autoimmune or vasculopathic forms of NSIP, highlighting the potential for a calibrated, phenotype-driven framework for molecular imaging in ILD where the [^18^F]FDG PET/CT metric of greatest prognostic value is selected according to disease biology rather than applied indiscriminately.
